# Factors affecting prescription of sodium-glucose co-transporter 2 inhibitors in patients with type 2 diabetes mellitus with established cardiovascular disease/ chronic kidney disease in Hong Kong: a qualitative study

**DOI:** 10.1186/s12875-022-01928-z

**Published:** 2022-12-07

**Authors:** Ngai Mui Ng, Yeung Shing Ng, Tsun Kit Chu, Phyllis Lau

**Affiliations:** 1 Department of Family Medicine and Primary Health Care, Tuen Mun Hospital, 23 Tsing Chung Koon Road, Tuen Mun, New Territories, Hong Kong SAR, China; 2 Department of General Practice, University of Melbourne, 780, Elizabeth Street, Melbourne, VIC 3010 Australia; 3 School of Medicine, Western Sydney University, Locked Bag 1797, Penrith, NSW 2751 Australia

**Keywords:** Type 2 diabetes, SGLT 2 inhibitors, Cardiovascular, Chronic kidney disease, Primary care, Qualitative study

## Abstract

**Background:**

Sodium-glucose co-transporter 2 inhibitors (SGLT2 I) has cardiorenal protective properties and are recommended for patients with diabetes and established atherosclerotic cardiovascular disease (ASCVD) and/or chronic kidney disease (CKD). Although cardiorenal complications are high in diabetes and pose a significant financial burden on the Hong Kong health care system, the use of SGLT2 I in these populations remains low. And yet this issue has not been explored in Hong Kong primary care. This study aimed to explore factors affecting primary care doctors’ prescribing of SGLT2 I in patients with diabetes and established ASCVD/CKD in Hong Kong.

**Methods:**

A phenomenological qualitative research using semi-structured interviews was conducted between January and May 2021 in one Hospital Authority cluster in Hong Kong. Purposive sampling was employed to recruit primary care doctors in the cluster. The Theoretical Domains Framework (TDF) underpinned the study and guided the development of the interview questions. Data was analysed using both inductive and deductive approaches. The Consolidated criteria for reporting qualitative research (COREQ) checklist was used to guide the reporting.

**Results:**

Interviews were conducted with 17 primary care doctors. Four overarching themes were inductively identified: knowledge and previous practice patterns influence prescription, balancing risks and benefits, doctors’ professional responsibilities, and system barriers. The four themes were then deductively mapped to the nine specific domains of the TDF: knowledge; intention; memory; beliefs about capabilities; beliefs about consequences; goals; role and identity; emotion; and environmental constraints. Most interviewees, to varying extent, were aware of the cardio-renal advantages and safety profile of SGLT2 I but are reluctant to prescribe or change their patients to SGLT2 I because of their knowledge gap that the cardio-renal benefits of SGLT2 I was independent of glyacemic efficacy. Other barriers included their considerations of patients’ age and renal impairment, and patients’ perceptions and preferences.

**Conclusions:**

Despite evidence-based recommendations of the utilisation of SGLT2 I in patients with established ASCVD/CKD, the prescription behaviour among primary care doctors was affected by various factors, most of which were amendable. Our findings will inform the development of structured interventions to address these factors to improve patients’ cardio-renal outcomes.

## Background

Diabetes management aims to prevent complications and maintain quality of life [1]. Atherosclerotic cardiovascular disease (ASCVD) and chronic kidney disease (CKD) remain the two most essential complications for diabetes worldwide. The crude incidence of diabetes complications in Hong Kong (HK) were 33.7% for albuminuria, 16.4% for stage 3 CKD or above, 6.1% for the history of coronary heart disease, and 5.1% for the history of stroke according to a recent territory-wide study [2]. ASCVD is one of leading causes of death and disability in diabetes [3]. Meanwhile, patients with CKD have a greater risk of cardiovascular complications [4], and CKD due to diabetes mellitus has become the major cause of end-stage renal failure (ESRF) requiring renal replacement therapy in HK [5]. These complications have posed a significant financial burden on the health care system. A local analysis on direct medical expenditure revealed a 5.94-fold increase in costs for new stroke events, and a 2 to 3-fold rise in expenses for acute myocardial infarction (MI), heart failure (HF), stroke, and ESRF [6].

Sodium-glucose co-transporter 2 inhibitor (SGLT2 I) belongs to a novel class of oral glucose-lowering agents. Besides glycaemic control, they have been shown to have other beneficial effects for the cardiovascular and renal systems, which include diuresis, reduction in weight, lowering of blood pressure [1, 7–9], as well as having low risks of hypoglycaemia [8]. They also have been proven to have substantial cardio-renal protective benefits, including a significant decrease in the composite outcome of MI, stroke, and cardiovascular death, reduced risk of cardiovascular mortality, reduction of all-cause mortality, reduction of hospitalisation for HF as well as progression of renal disease in four landmark clinical outcome trials [9–12] and real-world studies [13, 14]. The trials also suggested that the shown cardiovascular risk reduction effects were not mediated by glycaemic control [9–11]. The above findings have led to a significant shift from a sole emphasis on glycaemic control to improving cardio-renal outcomes when choosing glucose-lowering agents. SGLT2 I have been strongly recommended for patients with diabetes and established ASCVD /CKD/HF by the American Diabetes Association /European Association for the Study of Diabetes (ADA/EASD) guidelines [1], European Society of Cardiology (ESC) guidelines [15], and Kidney Disease: Improving Global Outcomes (KDIGO) guidelines [16]. Another glucose-lowering agent with cardio-renal protective benefits is glucagon-like peptide-1 receptor agonist (GLP-1 RA). It is however not available in primary care settings in HK and requires regular subcutaneous injections, which makes it less convenient for patients. As a result, the presence of established ASCVD and/or CKD is considered a compelling indication for utilising SGLT2 I in patients with diabetes in primary care settings in HK.

However, many factors can affect doctors’ decisions. A recent nationwide cohort study in the US found that only 7.2% pharmacologically treated patients with diabetes were prescribed SGLT2 I [17]. Additionally, there was a treatment-risk paradox that patients with MI and CKD were less likely to receive SGLT2 I [17]. One study in the UK showed that although the use of SGLT2 I was increasing, the overall utilisation of SGLT2 I remained low, and the prescription was irrespective of cardiovascular status [18]. Another study in the USA also revealed that very low utilisation of SGLT2 I among patients with diabetes and proteinuric CKD [19]. SGLT2 I was introduced in 2015 in HK. Although SGLT2 I is subsidised by the government and no extra out-of-pocket payment is needed from patients in public healthcare settings, the adoption rate of SGLT2 I by clinicians and patients were still low [20]. Therefore, there is great hesitancy in the utilisation of SGLT2 I both globally and locally.

Previous study suggested that clinical inertia, limited knowledge, insufficient treatment re-evaluation, cost and competing priorities may have contributed to the low prescription rates [21]. However, few studies have explored the influencing factors of prescription from the perspective of primary care doctors, and there was a lack of understanding of the underlying reasons for their prescription behaviour. Qualitative study helps to deeply explore peoples’ beliefs, experiences, and views [22]. In HK, greater than 65% patients with diabetes are managed in public settings [6]. Therefore, this study aimed to explore the factors affecting primary care doctors in the public healthcare setting in prescribing SGLT2 I for patients with diabetes and established ASCVD /CKD in HK using a qualitative approach. An increased understanding in primary care doctors’ decision-making process will help design effective and tailored improvement strategies to achieve better management of patients with diabetes in the public healthcare setting.

## Methods

### Study design

A phenomenological approach was adopted using in-depth semi-structured interviews to explore factors affecting primary care doctors on prescribing SGLT2 I in diabetic patients with established ASCVD/ CKD. The Consolidated Criteria for Reporting Qualitative Research (CQREQ) checklist was used to guide the reporting of this paper [23].

### Theoretical framework

Following a review of the literature on the different theories and frameworks available to guide the research, we adopted the Theoretical Domains Framework (TDF) because of its comprehensiveness in the determinants of behavioural change. The initial version of TDF was published in 2005, with a later version released in 2012 after validation [24]. It is a well-operationalised, multi-level determinant framework [24, 25]. It is composed of 14 theoretically domains that integrates constructs from 33 theories related to health behaviour modification (Table 1), and has been widely used in qualitative research to assess evidence-based implementation [26–29].

**Table 1:** Definition of the theoretical domains framework [22]

Domain	Definition
Knowledge	An awareness of the existence of something
Skills	An ability or proficiency acquired through practice
Social/professional role and identity	A coherent set of behaviours and displayed personal qualities of an individual in a social or work setting
Beliefs about capabilities	Acceptance of the truth, reality, or validity about an ability, talent, or facility that a person can put to constructive use
Optimism	The confidence that things will happen for the best, or that desired goals will be attained
Beliefs about consequences	Acceptance of the truth, reality, or validity about outcomes of a behaviour in a given situation
Reinforcement	Increasing the probability of a response by arranging a dependent relationship, or contingency, between the response and a given stimulus
Intentions	A conscious decision to perform a behaviour or a resolve to act in a certain way
Goals	Mental representation of outcomes or end states that an individual wants to achieve
Memory, attention and decision processes	The ability to retain information, focus selectively on aspects of the environment, and choose between two or more alternatives
Environmental context and resources	Any circumstance of a person’s situation or environment that discourages or encourages the development of skills and abilities, independence, social competence, and adaptive behaviour
Social Influences	Those interpersonal processes that can cause an individual to change their thoughts, feelings, or behaviours
Emotion	A complex reaction pattern, involving experiential, behavioural, and physiological elements, by which the individual attempts to deal with a personally significant matter or event
Behavioural regulation	Anything aimed at managing or changing objectively observed or measured actions

### Research team

NMN, YSN and TKC are primary care doctors working in the New Territories West region in HK. NMM (PhD) was an intermediate fellow undertaking her general practice training at the time of the study. YSN is supervisor of NMN; TKC has rich expertise in research. PL (PhD) is an experienced primary care qualitative researcher based at the University of Melbourne and Western Sydney University; PL instructed and mentored NMN in research methodology.

### Study setting

This research was conducted in the Department of Family Medicine and Primary Health Care in the New Territory West Cluster (NTWC), one of seven clusters of the Hospital Authority (HA) in HK. The research was designed to be conducted only in this cluster, where researcher NMN undertook this research as part of her general practice training.

The HA provides 80% of outpatient service for HK residents [2]. NTWC covers around 15% of total population of HK, and has eight General Out-patient Clinics (GOPCs) that serves about 55,000 patients with diabetes in year 2021.

### Sample

We employed a purposive sampling approach to recruit participants who had information to enable the exploration of factors that affect primary care doctors in the public healthcare setting in prescribing SGLT2 I for patients with diabetes and established ASCVD /CKD in HK.

### Sample selection criteria

A list of doctors working in the Department of Family Medicine and Primary Health Care in NTWC of HA in Hong Kong was collated. Inclusion criteria were doctors who were actively practicing in family medicine and managed patients with diabetes. Doctors who did not provide services for managing patients with diabetes were excluded. A total of 83 doctors were deemed to be eligible; one doctor was later excluded since her primary service group was paediatric patients.

### Recruitment

Recruitment occurred between January and May 2021. A matrix based on gender, age, years of practice and qualification, was used to select participants from the sample of 82 eligible primary care doctors to ensure maximum variation for the elicitation of comprehensive and diverse views. Selected primary care doctors were invited via text messages. Although participation was entirely voluntary, all invited primary care doctors agreed to participate. Participants were asked to sign an informed consent form before interviews were conducted. Selection and interview of participants continued to just beyond the point of data saturation as determined by the research team.

### Data collection

Each interview was conducted by researcher NMN in Cantonese, which was the native language of NMN and participants. She emphasised her role as a researcher and that it was not an official assessment of the interviewee’s knowledge before each interview started. Face-to-face interviews were conducted in a quiet location at participants’ workplace. Respondents’ demographic data was collected before interviews started.

Interview questions were developed based on the literature review and TDF (Table 2). There are six main open-ended and non-leading questions. Specific questions concerning the TDF domains were asked flexibly as probing and follow-up questions to clarify respondents’ views on them. The interview questions were piloted for comprehensiveness and comprehensibility with two primary care doctors who shared the same demographics and backgrounds as the target participants. Minor wording adjustments were made to optimise and finalise the interview guide (Table 2).

**Table 2:** Interview guide

	Interview questions (related TDF domains)
1.	Have you ever managed a diabetic patient with ASCVD and/ or CKD before? Could you please share your experience with me?
2.	Could you please tell me what you know about SGLT2 I? (Knowledge)
3.	What do you feel is your role in prescribing SGLT2 I? (Professional role and identity) - Prompt: As a family doctor, do you feel it is appropriate for you to prescribe SGLT2 I? - Follow up question: If you are to prescribe SGLT2 I to a patient, how confident or difficult would you feel? Can you tell me more? (Beliefs about capabilities) -Follow up question: In your opinion, what skills do you need to determine whether to prescribe SGLT2 I to a patient? (Skill)
4.	What factors may influence your decision on prescribing an SGLT2 I for a diabetic patient with ASCVD/CKD? (Memory) - Prompt: How to balance risks and benefits? (Beliefs about consequences) - Follow up question: Do you think you would prescribe SGLT2 I to a diabetic patient with established ASCVD/CKD? (Intentions) - Follow up question: Do you have any goals when prescribing SGLT2 I to diabetic patients with established ASCVD/CKD? (Goals) - Follow up question: Overall, how optimistic are you about using SGLT2 I in improving the prognosis of ASCVD and/or CKD? (Optimism) - Follow up question: Can you think of any situations that you would be concerned about prescribing SGLT2 I to a patient? (Emotion)
5.	What environmental factors would affect your prescription of SGLT2 I? Prompts: Are there clear guidelines?(resources) Any influence of Colleagues’ feedbacks / Supervisors’ feedbacks / Patients’ feedbacks? (Social Influences)
	How about the consultation time? (Environmental context)
	Is there any monitoring or feedback from the clinic on the use of SGLT2 I? (Reinforcement)
6.	Do you have any suggestions to improve the prescription of SGLT2 I? (Regulation)

All interviews were audio-recorded and transcribed verbatim by NMN. All interviews were anonymised, and field notes were made with permission from participants. No repeat interviews were carried out in the study. Interview transcripts were made available to the participants on request.

### Data analysis

Interview transcripts were imported into QSR Internationals NVivo Qualitative Data Analysis Software V.12. for management. The transcripts and fieldnotes were analysed independently and inductively by two researchers (NMN, YSN). The two coders had numerous iterative discussions to reconcile differences and reach consensus. Similar codes were organised into subthemes which were further organised into themes. Finally, overarching themes were allocated deductively into relevant TDF domains [29]. Discrepancies in coding and interpretation were discussed between NMN and YSN, with reflections on their potential biases and viewpoints, until consensus was reached. Salient quotes to support each theme were translated into English and back-translated to Cantonese to check for consistency.

### Ethical approval

The research protocol was approved by the HA NTWC Research Ethics Committee (ID no. NTWC/REC/20107) on 2 November, 2020.

## Results

Seventeen primary care doctors with diverse characteristics were interviewed (Table 3). The demographic information for individual participants is listed in Table 4. The interviews lasted from 29 min to 48 min (mean interview length 37 ± 6 min). Data saturation was reached after 13 interviews as determined by the research team. A further four interviews were carried out to verify that no more new themes emerged. These additional interviews did not contribute to the further development of new themes. No participants requested to read their transcripts.

**Table 3:** Participant demographics

Characteristics	Number of Participants
Gender	
Male	9
Female	8
Age	
≤ 30	4
31–40	4
41–50	5
> 50	4
Duration of Practice (Years)	
≤ 5	4
6–10	3
11–20	5
> 20	5
Family Medicine (FM) training status	
Non - FM training	3
Basic training	5
Intermediate fellows	4
FM specialist/SMO	5

**Table 4:** Individual information of the participants

	Gender	Age	Duration of practice	Family Medicine training status
A2	Female	31–40	≤5	Basic training
B2	Female	41–50	11–20	FM specialist/SMO
C6	Male	41–50	11–20	FM specialist/SMO
D2	Male	31–40	11–20	Basic training
D4	Male	31–40	6–10	Intermediate fellow
E3	Female	41–50	11–20	FM specialist/SMO
F2	Male	41–50	> 20	Non-FM training
G6	Male	≤30	≤5	Intermediate fellow
K7	Male	> 50	> 20	Non-FM training
N5	Female	≤30	≤5	Basic training
P9	Female	≤30	6–10	Intermediate fellow
Q7	Male	> 50	> 20	FM specialist/SMO
R5	Male	> 50	> 20	FM specialist/SMO
S3	Female	31–40	6–10	Basic training
T3	Female	≤30	≤5	Basic training
U12	Male	41–50	11–20	Intermediate fellow
W1	Female	> 50	> 20	Non-FM training

The inductive analysis generated four themes and nine subthemes that influenced doctors’ prescription. The four themes were: 1) Knowledge and previous practice patterns influence prescription; 2) Balancing risks and benefits; 3) Doctor’s professional responsibilities and 4) System barriers. These themes were then deductively mapped to the nine specific domains of the TDF. Theses TDF domains were: knowledge; intention; memory; beliefs about capabilities; beliefs about consequences; goals; role and identity; emotion; and environmental constraints. It was determined that TDF adequately captured all perceived determinants affecting utilisation of SGLT2 I. The themes, subthemes, related TDF domains, and the quotes to support them are displayed in Table 5. Participants were identified by unique codes.

**Table 5:** Quotes for each theme

Theme	Subtheme	TDF domain	Quote
Theme 1: Knowledge and previous practice patterns influence prescription	Subtheme 1: Awareness of cardio-renal benefits	knowledge	1: It is strongly recommended to be used in a patient with confirmed IHD…and renal involvement and proteinuria.(B2)
			2: I think it has a benefit, but I am not sure to what extent it can lower A1C or slow down the progression of renal impairment.(A2)
	Subtheme 2: Awareness of cardio-renal benefits independent of glycemic efficacy	knowledge	3: I’m not sure if it’s worth keeping these medications if there is no improvement in A1C (S3)
		goals	4: For most of my cases, I use it in suboptimal DM control. (D4)
		intention	5: Sometimes the diabetes control is so poor, such as the A1C level is already more than 10. In these cases, I thought prescribing this medication [SGLT2 I] would only help a little. (B2)
		intention	6: If the patient’s diabetes is well controlled, I will not prescribe this medicine because his A1C is already well-controlled. (P9)
	Subtheme 3: Perceived safety profile of SGLT2 I	memory	7: I think the good thing about SGLT2 is that it seldom has any side effects. For the most worrisome side effects, DKA, I have not seen once so far. Maybe it is rare. (T3)
		belief about capabilities	8: I rarely take the initiative to prescribe a new drug. (A2)
		intention	9: I have reservations about any new medicine, even though it has many benefits. I trust the evidence, but these patients are under my care for my practice, so I would prefer to be more cautious. (K7)
Theme 2: Balancing risks and benefits	Subtheme 1: Where benefits are obvious	beliefs about consequences	10: Many patients in our clinics have high A1C and high body weight. I think it is a good choice as it has a weight control benefit. (A2)
		intention	11: I would actively consider prescribing it if BP is suboptimal. (Q7)
	Subtheme 2: Concerns about use in the elderly	beliefs about consequences	12: Those older patients ...might have many complications and will not have too many days left. I prefer to use traditional methods to help them ..., so I will not prescribe this medicine to them. (K7)
		beliefs about consequences	13: My concern is people with old age. They may have many comorbidities… and not feel possible side effects. (D4)
		beliefs about consequences	14: Some older people live alone and get no visitors, .... I will be cautious as they might not understand what I say, therefore easily developing adverse effects.(E3)
	Subtheme 3: Concerns about use in patients with renal impairment	intention	15: I will only prescribe SGLT2 I for the patient with normal renal function with the creatinine level is not elevated. (P9)
		emotion	16: Some patients... have already developed renal impairment. I will be worried as I am unsure whether the renal function will gradually deteriorate to the level that I need to stop. (B2)
	Subtheme 4: Patients’ perceptions and preferences	beliefs about consequences	17: The patient is opposed to it. “I don’t like it. I don’t want to take so many medicines. I don’t think I have any problems. I am happy with my current status. “Then I have no methods. (F2)
		beliefs about consequences, emotion	18: Their usual reasons for refusing medications are: “I don’t think I have a problem.” “I am afraid of taking medicine.” “I am afraid of having adverse effects.” (U12)
Theme 3: Doctor’s professional responsibilities		role and identity	19: It is reasonable for us to prescribe this drug because, as family doctors, we manage many diabetic patients. (N5)
			20: I want to protect patients from recurrent cardiovascular events and slow down renal function deterioration, but I find it difficult to witness their outcome improvement. (T3)
Theme 4: System barriers	Subtheme 1: Clinic operation constraints	environmental constraints	21: Sometimes I feel a rush due to limited consultation time. (P9)
			22: I was unable to consult with the same patients. As a result, I am unable to monitor the medication’s impact. (W1)
	Subtheme 2: Cost	environmental constraints	23: We have to consider the cost in the Hospital Authority setting as it is not cheap, so it is better to use it if other drugs do not work. (D2)
			24: The most important concept is prevention is better than treatment of complications. In the end, the cost will be lower. (Q7)

### Theme 1: knowledge and previous practice patterns influence prescription

#### Subtheme 1: awareness of cardio-renal benefits

Most participants were generally aware of the cardio-renal benefits of SGLT2 I. They were aware of the evidence and the recommended use of SGLT2 I in patients with established ASCVD/CKD (Quote 1). However, some noted that they needed more specific data to enrich their knowledge concerning the concrete benefits of improved outcomes (Quote 2).

#### Subtheme 2: awareness of cardio-renal benefits independent of glycaemic efficacy

Although most participants were aware that SGLT2 I could improve cardiovascular and renal outcomes, they perceived the benefits resulted mainly from glycaemic improvement. There was a lack of understanding that the benefits were independent of glycaemic control (Quote 3). Many doctors still used glycated haemoglobin (HbA1c) as the main treatment targets (Quote 4) and most said they would not consider SGLT2 I when a patient’s HbA1c was high as they perceived its glucose-lowering effects to be suboptimal (Quote 5). Most interviewees preferred to keep the current regimen and not change to SGLT2 I in situations where the glycaemic control has already met the target (Quote 6).

#### Subtheme 3: perceived safety profile of SGLT2 I

Most participants perceived SGLT2 I as having a good safety profile (Quote 7). On the other hand, several participants reported uncertainty prescribing SGLT2 I due to its novelty (Quote 8). For these participants, although they acknowledged the guideline recommendations for SGLT2 I, they felt more comfortable using medications they were more familiar with and preferred to use SGLT2 I cautiously to avoid risks (Quote 9).

### Theme 2: balancing risks and benefits

#### Subtheme 1: where benefits are obvious

A variety of factors were considered by primary care doctors when making decisions about SGLT2 I, including patient characteristics, comorbid conditions, demographics, and social factors. In general, doctors acknowledged the many potential benefits of SGLT2 I including weight-losing effects, less hypoglycaemia, blood pressure lowering effects. Most participants expressed they were more willing to use SGLT2 I in the presence of obesity and suboptimal blood pressure control (Quote 10–11).

#### Subtheme 2: concerns about use in the elderly

Participants expressed many reservations about the use of SGLT2 I in the elderly population group. On the one hand, some participants believed that SGLT2 I had minor beneficial effects in survival improvement for the elderly patients, thus they preferred to be conservative when considering their patients’ limited life expectancy and multi-morbidity (Quote 12). On the other hand, they worried that their elderly patients could not managed SGLT2 I’s side effects due to multi-morbidity, physical frailty, communication deficits and limited social support (Quote 13–14).

#### Subtheme 3: concerns about use in patients with renal impairment

Renal impairment was another significant factor frequently reported as the main determinant for doctors’ decision-making. Despite the belief that SGLT2 I could improve renal outcomes and slow down the progression of diabetic kidney disease, most interviewees admitted that they still had hesitancy to utilise SGLT2 I in CKD patients. Some only use SGLT2 I if the renal function was within normal range (Quote 15). Some participants reported reluctance to use SGLT2 I in patients with renal impairment, as they worried that the renal function would deteriorate and fall outside the approved range for prescribing SGLT2 I and they had to cease it later (Quote 16).

#### Subtheme 4: patients’ perceptions and preferences

Patients’ perceptions and preferences were also frequently cited as obstacles to prescribe. Numerous barriers were identified, including patients’ lack of awareness of the importance of optimal disease control, lack of motivation due to the absence of symptoms, fear of adverse events, and medication avoidance (Quote 17–18).

### Theme 3: Doctor’s professional responsibilities

Participants acknowledged the importance of their role for good glycaemic control and secondary prevention in patients. Most respondents commented that it was appropriate for primary care doctors to initiate SGLT2 I (Quote 19). Some doctors thought the benefits of SGLT2 I on secondary prevention was less apparent than its benefits on HbA1c that may occur within a shorter period of time (Quote 20).

### Theme 4: system barriers

#### Subtheme 1: clinic operation constraints

Consultation time was recognised as one significant element affecting doctors’ ability to discuss a new medication with patients and therefore directly impact prescription (Quote 21). Lack of continuity of care was also a factor. Some participants said they might not provide follow-up for the same patients and so may not be able to witness the beneficial effects of SGLT2 I (Quote 22).

#### Subtheme 2: cost

Participants raised two different views concerning cost. Some participants expressed concerns about cost issues in a public setting. They suggested that this newer and more expensive drug be used cautiously to prevent overrunning the budget (Quote 23). Other participants believed the potential long-term benefits such as reducing admission outweighed the immediate prescribing costs (Quote 24).

## Discussion

To our knowledge, this is one of few studies to apply a qualitative method to assess factors influencing primary care doctors’ prescriptions of SGLT2 I for patients with diabetes and ASCVD/CKD. Using TDF as a theoretical framework and in-depth semi-structured interviews, we were able to obtain a comprehensive and systematic understanding of the determinants affecting doctors’ prescriptions.

Generally, most participants were familiar with the cardio-renal advantages of SGLT2 I and believed it had a good safety profile. They also recognised their role in secondary prevention. However, there were a few roadblocks to prescription. Some were unique to SGLT2 I, others were common barriers encountered in general.

The most prominent barrier found in our study was a lack of understanding that the cardio-renal benefits of SGLT2 I was independent of glycaemic control. Despite acknowledging the established value and effectiveness of SGLT2 I in cardio- renal outcomes trials, most participants still prescribed it purely as a hypoglycaemic agent, ignoring this crucial tool for cardio-renal protection. Most doctors would not consider adopting SGLT2 I if the HbA1Cc was too high or within the target. This prescribing pattern reflects a lack of knowledge about updated guidelines. A recent survey also revealed a similar knowledge gap among cardiologists [30], which showed the top barrier for prescription of SGLT2 I was a lack of knowledge. Furthermore, more than half of the interviewed cardiologists did not feel it was their responsibility to prescribe anti-diabetic medications [30]. A previous qualitative study has demonstrated similar knowledge gap of under-appreciation of the cardio-renal benefits of SGLT I by general practitioners, contributing to low prescription rates [31]. However, in contrast to their findings that there was a preference for endocrinologists to initiate therapy, most of our interviewees thought it was appropriate for primary care doctors to initiate SGLT2 I. A possible explanation for the difference could be the fact that our participants were experienced in diabetes management as the majority of patients with diabetes in NTWC were under the care of the public sector. This was also echoed by the same study that general practitioners who frequently managed diabetes were more confident to prescribe SGLT2 I for patients with diabetes [31]. There is, however, a great need to enhance our frontline primary care doctors to use SGLT2 I more actively for their cardiorenal protective effects, and not just for glycaemic control.

Another main concern was renal impairment. Despite knowing the renal protective effects of SGLT2 I, many participants were reluctant to use in patients with renal impairment. This phenomenon was also observed in a cross-sectional study in Korea which showed the utilisation of SGLT2 I was significantly higher in CKD patients with better estimated Glomerular Filtration Rate (eGFR) than those with lower eGFR [32]. A recent study in the UK showed that when SGLT2 I was initiated, over 90% of patients had an eGFR≥60 mL/minute/1.73 m^2^, while only 1.7% had an eGFR < 60 mL/minute/1.73 m^2^ [33]. This indicates that the prescription of SGLT2 I was heavily affected by patients’ renal impairment. There were also some conflicting recommendations regarding the use of SGLT2 I in patients with CKD which may have led to hesitations in primary care doctors prescribing SGLT2 I. The approved renal threshold for eGFR for prescription of SGLT2 I was 45 mL/minute/1.73 m^2^ at the time (January to May 2021) of this study. However, this threshold had been reduced to 30 mL/min/1.73 m^2^ in late 2021 in HK by the pharmaceutical company following the emergence of evidence. With the results from the DAPA-CKD trials [34] and other ongoing clinical trials involving the administration of SGLT2 I in different CKD stages, the authorised renal threshold for starting SGLT2 I might even be lower. Therefore, primary care doctors need to update regularly with the latest guideline recommendations. Future strategies should be in place to address some of the common individual (including a lack of awareness of the evidence), health system (including time constraints), and contextual barriers (including a lack of agreement with the evidence) for the implementation of clinical practice guidelines [35].

Moreover, patients’ age was recurrently cited as a critical factor affecting doctors’ decision-making. Old age has always been a key concern in pharmacotherapy due to their medical complexity, multi-morbidity, frailty, and the risk of polypharmacy [36]. For example, the use of statin has been reported to be suboptimal in elderly aged 65 to 79 years with cardiovascular disease, despite recommendations by multiple international guidelines and decades of clinical evidence [37]. For SGLT2 I, the cardio-renal benefits were consistent across all age groups including those over 65 [38, 39]. The post hoc analysis of the EMPA-REG OUTCOME study, which included 35.3% of patients between 65 and < 75 years old and 9.3% ≥75 years old, found that empagliflozin reduced the risks of CV mortality, heart failure, and renal outcomes in all age groups [38]. Similarly, post hoc analysis of the DECLARE study, in which 40% of patients were between 65 and 75 years old, and 6% > 75 years old, found that dapagliflozin is effective and safe for all ages [40]. Additional efforts are still required nonetheless to address the ongoing concerns of safety of SGLT2 I in the older age groups and give confidence to doctors prescribe SGLT2I in this population.

This study also revealed that organisational constraints, such as time constraints and lack of continuity of care, and patients’ perceptions and preferences, were barriers affecting prescription of new medications. This had been echoed in the findings from prior studies on guideline adherence [41].

Based on the findings of this study, several strategies could be implemented to improve the prescription of SGLT2 I in patients with diabetes and established ASCVD/CKD. Firstly, doctors should have additional training and education for emphasising a paradigm shift away from only glycaemic management and toward cardio-renal protection, which is crucial for implementation of the evidence-based guidelines. An open and ongoing process is needed to encourage doctors to voice their concerns. Secondly, patients should have more education on cardio-renal risk control. This could be achieved through a patient empowerment program and supported by a multidisciplinary team, which has been proved to be able to reduce the incidence of diabetic complications, hospitalisations, and mortality [42]. Finally, for policymakers, they can add the prescription of SGLT2 I as a “key performance indicator” besides HbA1c control in patients with diabetes and established ASCVD/CKD. The latest ADA guidelines have suggested using SGLT2 I in this populationindependent of HbA1c status [43]. Since the HA maintains computerised data in the Clinical Management System for all patients under its care, policymakers can readily monitor the utilisation of SGLT2 I and establish objectives to gradually increase the adoption rate of SGLT2 I in patients with established ASCVD/CKD.

### Strengths and limitations of this study

A key strength of the study is the use of qualitative methods to explore in-depth the beliefs, views and experience of primary care doctors in prescribing SGLT2 I. However, the study was conducted in only one cluster in the public setting of HK. Findings may not be extrapolatable to other settings, such as outpatient clinic in other regions or private setter. Despite efforts made to ensure a broad representation of participants, selection bias was possible due to the small number. Future studies should involve participants from different primary care settings including public and private sectors. Furthermore, although there are many other possible indications for use of SGLT2 I, we only focused on the behaviour of prescription of SGLT2 I in patients with diabetes and established ASCVD /CKD in our study, because these were the two most common complications that are managed in our setting. However, our findings may be extrapolated to other related prescription behaviour and inform other change strategies.

## Conclusions

Our study suggests that there may be a significant knowledge gap among primary care doctors, and providers’ prescriptions are influenced by many factors, especially consideration of patients’ age, renal impairment, and patients’ perceptions and preferences. Our findings highlight the need for further interventions in HK’s public primary health care sector to address these factors to improve patients’ cardio-renal outcomes in the future.

## Data Availability

The datasets used and/or analysed during the current study available from the corresponding author on reasonable request.

## Abbreviations

ASCVD: Atherosclerotic cardiovascular disease
CKD: Chronic kidney disease
HK: Hong Kong
ESRF: End-stage renal failure
MI: Myocardial infarction
SGLT2 I: Sodium-glucose co-transporter 2 inhibitor
TDF: Theoretical Domains Framework
HA: Hospital Authority
NTWC: New Territory West Cluster
